# Novel Pyrimidine Derivatives as Antioxidant and Anticancer Agents: Design, Synthesis and Molecular Modeling Studies

**DOI:** 10.3390/molecules28093913

**Published:** 2023-05-05

**Authors:** Malama Myriagkou, Evangelia Papakonstantinou, Georgia-Eirini Deligiannidou, Alexandros Patsilinakos, Christos Kontogiorgis, Eleni Pontiki

**Affiliations:** 1 Department of Pharmaceutical Chemistry, School of Pharmacy, Faculty of Health Sciences, Aristotle University of Thessaloniki, 54124 Thessaloniki, Greece; myriagkou@pharm.auth.gr; 2 Laboratory of Hygiene and Environmental Protection, School of Medicine, Democritus University of Thrace, 25510 Alexandroupoli, Greece; evangeliapapakonstantinou7@gmail.com (E.P.); edeligia@med.duth.gr (G.-E.D.); ckontogi@med.duth.gr (C.K.); 3 Department of Drug Chemistry and Technology, Sapienza University, 00185 Rome, Italy; alexandros.patsilinakos@gmail.com

**Keywords:** antioxidant, anti-inflammatory, cytotoxicity, HaCaT, pyrimidine derivatives, LOX inhibition

## Abstract

The heterocyclic ring system of pyrido [2,3-*d*]pyrimidines is a privileged scaffold in medicinal chemistry, possessing several biological activities. The synthesis of the pyrimidine derivatives was performed via the condensation of a suitable *α,β*-unsaturated ketone with 4-amino-6-hydroxy-2-mercaptopyrimidine monohydrate in glacial acetic acid. Chalcones were synthesized, as starting materials, via the Claisen–Schmidt condensation of an appropriately substituted ketone and an appropriately substituted aldehyde in the presence of aqueous KOH 40% *w/v* in ethanol. All the synthesized compounds were characterized using IR, ^1^H-NMR, ^13^C-NMR, LC-MS and elemental analysis. The synthesized compounds were evaluated for their antioxidant (DPPH assay), anti-lipid peroxidation (AAPH), anti-LOX activities and ability to interact with glutathione. The compounds do not interact significantly with DPPH but strongly inhibit lipid peroxidation. Pyrimidine derivatives **2a** (IC_50_ = 42 μΜ), **2f** (IC_50_ = 47.5 μΜ) and chalcone **1g** (IC_50_ = 17 μM) were the most potent lipoxygenase inhibitors. All the tested compounds were found to interact with glutathione, apart from 1h. Cell viability and cytotoxicity assays were performed with the HaCaT and A549 cell lines, respectively. In the MTT assay towards the HaCaT cell line, none of the compounds presented viability at 100 μM. On the contrary, in the MTT assay towards the A549 cell line, the tested compounds showed strong cytotoxicity at 100 μM, with derivative **2d** presenting the strongest cytotoxic effects at the concentration of 50 μΜ.

## 1. Introduction

Inflammation is a physiological immune system’s response to harmful stimuli, such as infection, injury, irradiation or toxic compounds, aiming to eliminate the initial cause of cell injury and initiate the healing process [1]. Inflammation can be classified as either acute or chronic. During the initiation and resolution of the inflammatory response, the coordinated expression of many factors takes place, including growth factors, cytokines, chemokines, oxidative stress products, lipid mediators and proteases [2]. Acute inflammation is characterized by redness, swelling, heat and pain and is usually of short duration, depending on the severity of the injury. Although inflammation is a defense mechanism vital to health, persistent induction and dysregulation can lead to chronic inflammation [3]. Chronic inflammation is associated with various diseases, such as asthma, cancer, diabetes, rheumatoid arthritis, cardiovascular disease and Alzheimer’s disease [4].

A possible link between inflammation and cancer was first proposed in 1863 by Rudolf Virchow, who observed the presence of leukocytes in neoplastic tissues. The critical role that inflammation plays in tumorigenesis was not widely understood until the last decade, when some underlying molecular mechanisms were elucidated [5]. Epidemiological studies have revealed that chronic inflammation makes individuals susceptible to several types of cancer [6]. Recent studies have shown that the microenvironment of inflammation can induce the initiation of tumors and is involved in various steps of tumorigenesis, including survival, proliferation, invasion, angiogenesis and metastasis [7].

**Figure 1:**
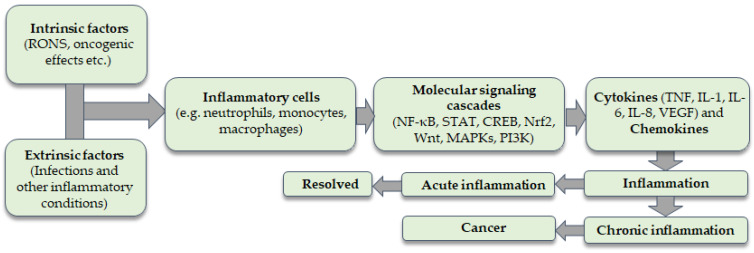
Intrinsic and extrinsic factors provoke the recruitment of inflammatory cells to the site of inflammation. Molecular signaling cascades are activated which are related to the coordinated expression of inflammatory cytokines. Inflammation can be acute or chronic. Chronic inflammation is linked with an increased risk of carcinogenesis.

The most important features of cancer-related inflammation include leukocyte infiltration, mainly macrophage and cytokine expression, such as TNF-*α*, IL-1 and IL-6, or chemokines such as CCL2, tissue remodeling and angiogenesis [8]. The link between inflammation and cancer can be considered as consisting of two pathways. The intrinsic pathway is associated with genetic events, such as oncogenes or genetic aberrations, causing inflammation and neoplastic transformation. Inflammatory cells and mediators are components of the microenvironment of most tumors and, thus, are present in cases for which there is no epidemiological basis for inflammation. In the extrinsic pathway, inflammatory or infectious conditions augment the risk of developing cancer [9] (Figure 1).

Carcinogenesis can be induced by inflammation through the production of reactive oxygen species (ROS) and reactive nitrogen species (RNS) [10]. Inflammation activates a variety of inflammatory cells, such as neutrophils and macrophages, which are able to produce ROS and RNS through the activation of oxidant-generating enzymes, such as NADPH oxidase, xanthine oxidase, inducible nitric oxide synthase (iNOS) and myeloperoxidase. These species are produced in order to eliminate the initial cause of induced inflammation. However, they can potentially cause cellular damage to nucleic acids, lipids and proteins by oxidation, nitration, chlorination and bromination reactions, leading to mutagenesis and altered functions of proteins [11]. The most common point mutations induced by oxidative DNA damage include G:C to A:T transitions, followed by G:C to T:A [12].

**Figure 2:**
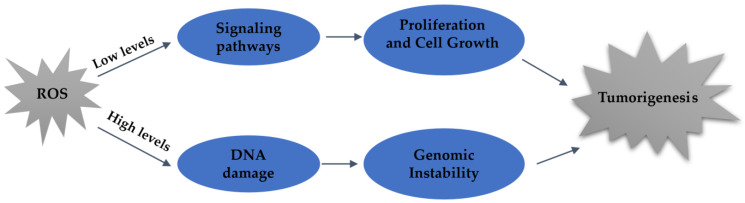
ROS levels determine cellular outcomes. The induction of ROS at lower levels leads to the activation of signaling pathways responsible for regulating cellular proliferation and growth. Conversely, high levels of ROS generation can lead to DNA damage, resulting in genomic instability and tumorigenesis.

Furthermore, lipoxygenases are dioxygenases playing a key role in inflammation. Lipoxygenases metabolize arachidonic acid to pro-inflammatory mediators, known as leukotrienes, or anti-inflammatory mediators, known as lipoxins. These lipid mediators are implicated in activating pro-inflammatory signal transduction pathways, such as the nuclear factor κB (NF-κB) pathway linking lipid signaling and transcription factor activation [13] (Figure 2).

Compounds presenting antioxidant and anti-lipoxygenase might have both therapeutic and prophylactic effects on cancer. Especially for the treatment of multidrug-resisting tumors, there is an extreme need for molecules presenting different activities interfering with multiple cancer-related pathways. Pyrido [2,3-*d*]pyrimidine is a privileged heterocyclic scaffold in drug design. Pyrido [2,3-*d*]pyrimidine ring systems have several biological and pharmacological activities, such as being analgesic, anti-inflammatory [14,15,16,17], anticancer [18], antitubercular [19], antimicrobial [20,21,22], a threonine tyrosine kinase (TT) inhibitor [23], an adenosine kinase inhibitor [24], a dihydrofolate reductase inhibitor [25,26], antiviral [27] and antioxidant [28,29,30].

Over the last decades, the treatment of multi-factorial diseases such as cancer has been based on multi-target-directed-ligand practices. Multifunctional compounds (MFCs) are broadly classified as hybrid and chimeric drugs combining two or more or merged pharmacophore groups allowing binding to two or more targets [31]. The combination of two or more or overlapped pharmacophore groups on the same scaffold leads to the rational design of new “smart” molecules maintaining pre-selected characteristics of the original templates, illustrating some benefits as well, e.g., reduced risk of toxicity and adverse reactions.

**Figure 3:**
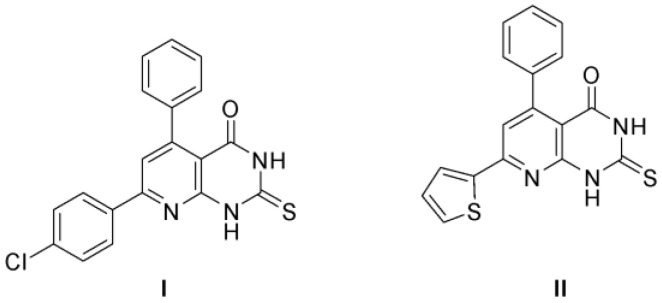
Substituted pyrido [2,3-*d*]pyrimidine derivatives as antimicrobial and anticancer agents [32,33].

Fares et al. have studied different substituted pyrido [2,3-*d*]pyrimidine derivatives as antimicrobial and anticancer agents [32,33]. Compound I has been extensively studied for antimicrobial activity, while compound II derivatives have been evaluated for anticancer activity (Figure 3).

Herein, based on these findings for anticancer activity of pyrimidine derivatives, bioisosteric replacements to the main scaffold (II) of the already published anticancer compounds are made, aiming to achieve an enhanced activity. Additionally, molecular docking studies on soybean lipoxygenase and drug-likeness for a library of substituted pyrido [2,3-*d*]pyrimidine derivatives guided the design and the synthesis of the novel derivatives with multiple biological activities. The synthesized eight pyrido [2,3-*d*]pyrimidine derivatives have been structurally characterized and in vitro evaluated as novel lipoxygenase (LOX) inhibitors, with potential antioxidant and anticancer activity. The interaction with the stable free radical 2,2-diphenyl-1-picrylhydrazyl (DPPH), 2,2′-Azobis(2-methylpropionamidine) dihydrochloride (AAPH)-induced lipid peroxidation inhibitory activity, soybean lipoxygenase inhibitory activity and interaction with glutathione is assessed, and their cell viability in human epidermal keratinocyte cells (HaCaT) and cytotoxicity in adenocarcinomic human alveolar basal epithelial cells (A549) is studied.

## 2. Results and Discussion

### 2.1. Chemistry

**Scheme 1:**
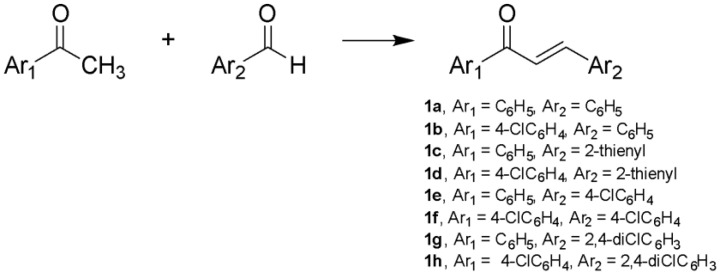
Synthesis of *α,β*-unsaturated ketones (**1a**–**1h**) using: EtOH, aqueous KOH 40% *w/v*, 0 °C, 3 h.

**Scheme 2:**
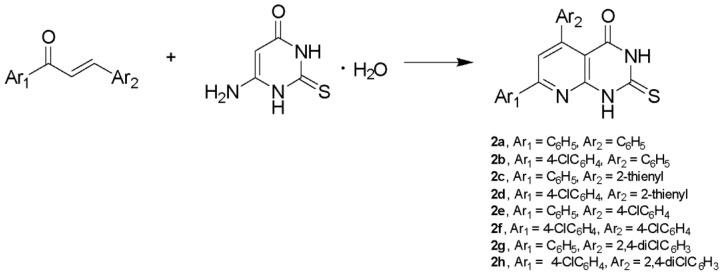
Synthesis of pyrimidine derivatives (**2a**–**2h**) using: glacial acetic acid, 118 °C, 96 h.

The synthesis of *α,β*-unsaturated ketones (**1a**–**1h**), as starting materials, was successfully accomplished *via* Claisen–Schmidt condensation between an appropriately substituted ketone and an appropriately substituted aldehyde in absolute ethanol in the presence of aqueous KOH 40% *w/v*, as shown in Scheme 1 [34] (Scheme 1). Pyrimidine derivatives (**2a**–**2h**) were synthesized *via* the condensation between suitable *α,β*-unsaturated ketones and 4-amino-6-hydroxy-2-mercaptopyrimidine monohydrate in glacial acetic acid (Scheme 2) [35].

Compounds **2a**–**2h** (Table 1) were obtained in moderate yields (14–65%). The final products were purified by column chromatography, preparative thin-layer chromatography or recrystallization from ethanol or ethyl acetate, while *α,β*-unsaturated ketones were recrystallized from ethanol. Compounds **2a**, **2b**, **2d**, **2e** and **2f** have been previously reported [16,33,35,36,37]. IR spectra for pyrimidine derivatives revealed the presence of a C=N bond at 1555 cm^−1^, C=O bond at 1670–1690 cm^−1^, C=S bond at 1170–1177 cm^−1^ and two N-H bonds at 3100–2900 cm^−1^. In ^1^H-NMR spectra, the final products gave three characteristic peaks: i) a singlet at δ 7.50 ppm assignable to H6 of the pyridine ring and ii) two broad peaks at 12–13 ppm assignable to the N-H protons of the pyrimidine ring. In ^13^C-NMR spectra, thiocarbonyl carbon resonates at 175 ppm and carbonyl carbon resonates at 160 ppm. Moreover, LC/MS and elemental analysis were carried out to confirm the novel derivatives. The physical data of the synthesized compounds are given in detail in the Experimental Section.

**Scheme 3:**
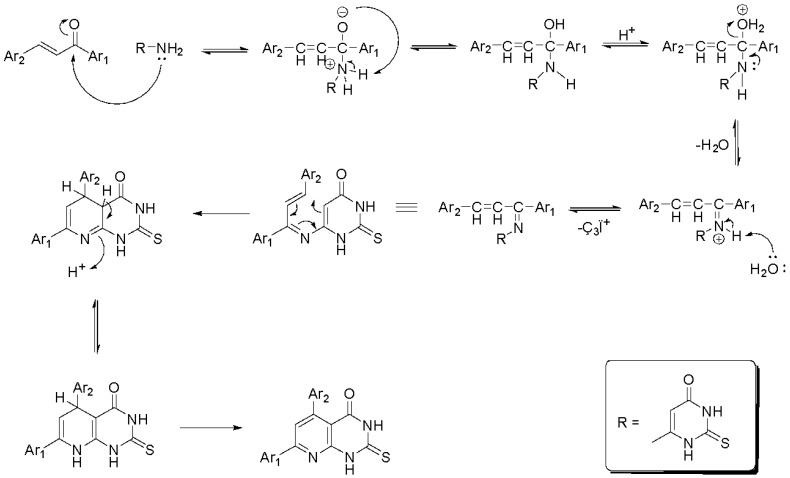
Reaction mechanism of the synthesized pyrimidine derivatives.

The reaction mechanism is a heterocyclic condensation of the uracil amino group with the propenone carbonyl **1a**–**1h** to the corresponding Schiff bases. It is a conjugate addition, accompanied by fragmentation and followed by multistep reactions to the final 1,2-addition product. Then, an intramolecular cyclisation takes place, providing the 5,6-dihydropyrido [2,3-*d*]pyrimidines, which are further tautomerized to the 5,8-dihydropyrido [2,3-*d*]pyrimidines and finally oxidized (Scheme 3) [32,33,35,37]. It has been found that with condensation of 6-amino-thio-uracil with *p*-substituted chalcones and Ar_1_ or Ar_2_ having electron-withdrawing groups (e.g., Cl, NO_2_), the oxidized product is obtained.

### 2.2. Physicochemical Studies

#### 2.2.1. Determination of Lipophilicity

Lipophilicity is an important physicochemical parameter of a drug candidate, linking solubility and membrane permeability with the ADMET (absorption, distribution, metabolism, elimination and toxicity) properties and the overall suitability of a molecule. Reversed-phase thin-layer chromatography was the method used to experimentally determine the lipophilic character of the synthesized compounds as R_M_ values (Table 2) [38]. Lipophilicity was also theoretically calculated as clog *P* values using the Bio-Loom program of BioByte Corp (http://www.biobyte.com/, (accessed on 1 March 2023)). An attempt was made to correlate the experimental R_M_ values with the theoretically calculated clog *P* values (Table 2). Nevertheless, this attempt proved unsuccessful. Both the *α,β*-unsaturated ketones (**1a**–**1h**) and the pyrimidine derivatives (**2a**–**2h**) showed low lipophilicity as R_M_ values (negative scale), whereas the calculation indicated higher lipophilicity. This disagreement could be attributed to several factors, e.g., different solvation, silanophilic interactions and hydrogen bonding.

#### 2.2.2. Theoretical Calculation of Physicochemical Properties

Drug-likeness is a qualitative estimation of how a molecule could become an oral drug with respect of bioavailability. This assessment can be made from the molecular structure, even before the molecule’s synthesis and its biological evaluation. Lipinski’s Rule of Five is usually used to evaluate drug-likeness. The synthesized compounds were analyzed in silico using the online platform Molinspiration (Molinspiration Cheminformatics 2023, www.molinspiration.com, (accessed on 1 March 2023)). The results are presented in Table 3.

### 2.3. Biological Evaluation

Oxidative stress is defined as the imbalance between the production of free radicals and the antioxidant defense mechanisms and is associated with many pathophysiological conditions, such as neurodegenerative diseases and cancer. Antioxidants, which can be exogenous or endogenous, enzymatic or non-enzymatic, can delay or inhibit cellular damage, mainly through their free radical scavenging activity [39].

In this study, the synthesized compounds were evaluated in vitro for their ability to interact with the stable free radical DPPH, inhibit the AAPH-induced linoleic acid peroxidation, inhibit soybean lipoxygenase and interact with glutathione. Moreover, the MTT assay was used to assess the cell viability and cytotoxicity in the presence of the synthesized compounds.

All the synthesized compounds were studied for their ability to interact with the stable free 2,2-diphenyl-1-picrylhydrazyl radical (DPPH) at a concentration of 100 μM after 20 min and 60 min (Table 4). This assay is based on the reduction of DPPH by transferring one electron from the antioxidant. Nordihydroguaretic acid (NDGA) was used as a reference compound [40]. Generally, the compounds presented low or moderate reducing activity. In particular, the activity of chalcones **1a** and **1c** seemed to decrease over time. The reducing activity of derivatives **2a** and **2e** increased over time, whereas the activity of derivative **2c** decreased over time. The scavenging activity of the compounds seems to correlate with low lipophilicity values. Both chalcones **1a** (clog *P* = 3.08), **1c** (clog *P* = 2.73) and the corresponding pyrimidine derivatives **2a** (clog *P* = 3.64), **2c** (clog *P* = 3.53) were characterized by low lipophilicity values. An exception is compound **2e**. Pyrimidine derivatives **2a**, **2c** and **2e,** which presented some reducing activity, were characterized by low-molecular-weight volume values, which favors the approach of the bulky radical DPPH. Furthermore, the kind of aromatic ring seems to affect activity. The presence of thienyl instead of phenyl ring reduced the interactions with DPPH radical, both in chalcones and the corresponding pyrimidine derivatives.

For the lipid peroxidation study, 2,2′-Azobis(2-methylpropionamidine) dihydrochloride (AAPH) was used as the generator of peroxy radicals and sodium linoleate as the substrate that undergoes peroxidation. The conversion of linoleic acid to 13-hydroperoxy-linoleic acid was recorded at 234 nm. The change of absorbance reflects the possible antioxidant activity of the tested compounds, in comparison with the reference compound, Trolox. With the exception of compounds **1c** and **2f**, which showed no activity in our experimental conditions, the inhibitory activity of chalcones ranged from 2–51%, while pyrimidine derivatives presented high inhibitory activity (33–99%). The most potent compounds were compounds **2g** and **2h** (Table 4). Lipophilicity seems to favor the lipid peroxidation inhibition both in chalcones and in pyrimidine derivatives. The substitution of phenyl ring from the thienyl ring led to a decrease in inhibition in the case of chalcones (compounds **1a**, **1c** and **1b**, **1d**). The same substitution did not seem to affect activity in the case of pyrimidine derivatives (compounds **2a**, **2c** and 2**b**, 2**d**).

Lipoxygenase (LOX) is the key enzyme in the biosynthesis of inflammatory mediators; thus, it is considered a target for inflammatory diseases. Soybean LOX is a plant enzyme that has been used widely as a prototype for studying the functional and structural properties of the homologous family of lipoxygenases [41], presenting satisfactory homology with the human 5-LOX [42]. Linoleic acid is the most common substrate in plant LOX. In this study, all the synthesized compounds were evaluated as LOX inhibitors, indicating anti-inflammatory activity (Table 4). The most potent derivatives among pyrimidine derivatives were found to be **2a** (IC_50_ = 42 μM) and **2f** (IC_50_ = 47.5 μM), while a potent chalcone **1g** (IC_50_ = 17 μM) was also found. Lipophilicity is considered one of the most important parameters with regard to LOX inhibitors according to the literature [43]. On the contrary, in our study, molecular volume seems to affect the inhibitory activity of pyrimidine derivatives. Indeed, the most potent pyrimidine derivative **2a** (MV = 283.51) is characterized by a lower molecular volume value than derivative **2f** (MV = 310.58).

Alkylating agents, which can selectively react with free sulfhydryl groups in proteins in the body, could be used as anticancer drugs, overriding the adverse effects (e.g., mutagenesis) that stem from their reaction with hydroxyl or amino groups [44]. In this protocol, glutathione was used as thiol standard. The interaction of the tested compounds with glutathione is an indication of their potential anticancer activity. It is known from the literature that *α,β*-unsaturated ketones form Michael adducts while reacting with glutathione. In this study, the pyrimidine derivatives also included to find out any possible interaction with glutathione. All the tested compounds presented the ability to interact with glutathione after incubation, apart from compound **1h**. Compounds **2a** and **2f** were found to have the strongest interaction with glutathione, which can be used to further investigate their anticancer activity (Table 5).

The MTT (3-(4,5-dimethylthiazol-2-yl)-2,5-diphenyltetrazolium bromide) assay is a colorimetric method that is used for the evaluation of cell viability. MTT is converted into colored formazan crystals, indicating the activity of mitochondria and therefore the viability of the cells. Unlike dead cells, the metabolic processes of living cells are functional, meaning that the dehydrogenase enzymes are able to reduce the yellow MTT salt solution to purple crystals, at 37 °C. The intensity of the sample’s color, due to the amount of formazan crystals, is proportional to the number of the living cells in it. Additionally, the colored product is dissolved in various solubilizing agents, such as dimethyl sulfoxide (DMSO). The colored products can be quantitatively measured at 450 nm with a plate reader spectrophotometer [45]. In this study, the human epidermal keratinocyte cell line (HaCaT) was treated with different concentrations (100 μM, 50 μM, 10 μM and 1 μM) of the tested compounds and then the viability rates at each effect were calculated. These effects caused an increase in cell proliferation or led to cell death at different concentrations. The viability results are presented in Table 6.

The tested compounds showed no cell viability at the 100 µM concentration, although some of them showed moderate viability rates at the concentration of 50 μΜ, which increased at the concentration of 10 μM. Furthermore, the effect of compound **2f** was viable only at the concentration of 1 μΜ, with a percentage of 73.4%.

Accordingly, ΜΤΤ assay can be used to measure *in vitro* cytotoxic effects of different compounds in cancer cell lines, such as the adenocarcinoma human alveolar basal epithelial cell line (A549). In this case, the more cytotoxic properties a substance has, the fewer formazan crystals are formed and the less viability is finally detected. The compounds of this study were also tested for their cytotoxic effects against the adenocarcinomic human alveolar basal epithelial cell line (A549) at different concentrations (100 μM, 75 μΜ, 50 μM and 10 μM). The results are presented in Table 7.

Most of the derivatives showed strong cell toxicity at the 100 µM concentration compared to the positive control (silibinin). Only chalcones **1c**, **1e** and **1g** had low cytotoxicity rates at the same concentration, which were further reduced at 50 µM. All the other compounds presented moderate to high cytotoxicity against A549 cells, at the concentrations of 75 and 50 μM, with derivative **2d** showing the strongest cytotoxic effects (100%). Finally, the cells treated with the concentration of 10 µM showed moderate cytotoxicity with rates ranging around 40–60%.

### 2.4. Computational Studies—Docking Simulation Soybean Lipoxygenase

All the synthesized pyrimidine derivatives and the chalcones were subjected to in silico studies to soybean lipoxygenase-1 ( 3PZW) being in accordance with the biological protocol. Lipoxygenases contain a ‘‘non-heme’’ iron per molecule catalyzing the oxygenation of free and esterified polyunsaturated fatty acids containing a (1*Z*, 4*Z*)-penta-1,4-diene system to the corresponding hydroperoxides. Lately, it has been discovered that lipoxygenases present apart from the substrate-binding site (iron-binding site) in potential allosteric binding sites [46,47,48]. All the chalchones and the pyrimidine derivatives have been studied for their binding mode to the active site and to the whole protein so as to encompass all the allosteric sites. The synthesized compounds seem to bind to soybean lipoxygenase in an allosteric way, not entering in the enzyme’s cavity. Previous publications confirm these findings [47,49,50]. The docking scores of all the compounds, expressed in kcal/mol, are shown in Table 8; the lower the docking score is, the stronger/the better the binding affinity.

**Figure 4:**
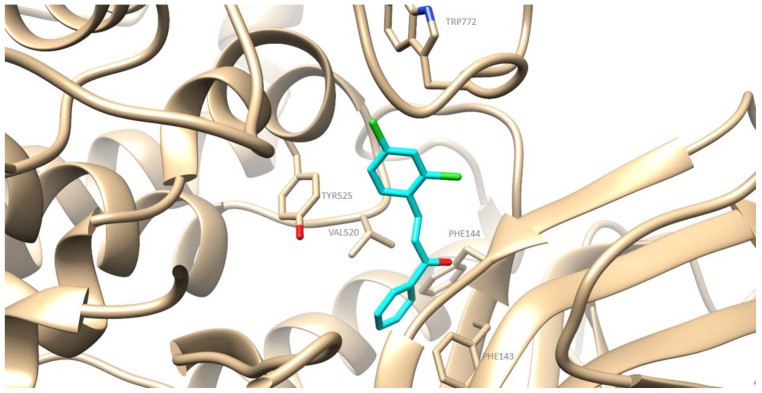
Preferred docking pose of **1g** (depicted in cyan) bound to soybean lipoxygenase (LOX-1).

**Figure 5:**
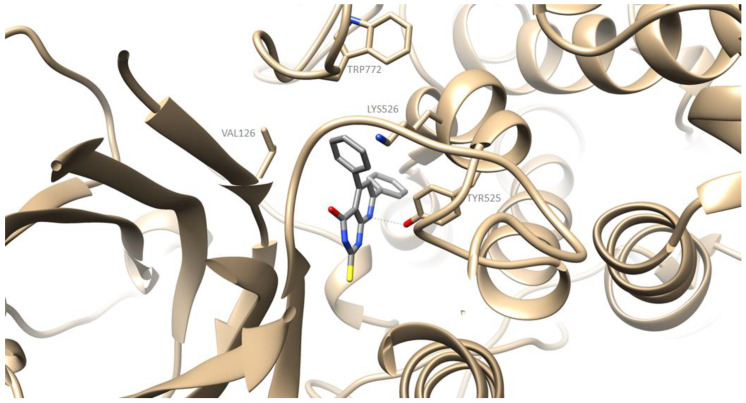
Preferred docking pose of **2a** (depicted in grey) bound to soybean lipoxygenase (LOX-1).

**Figure 6:**
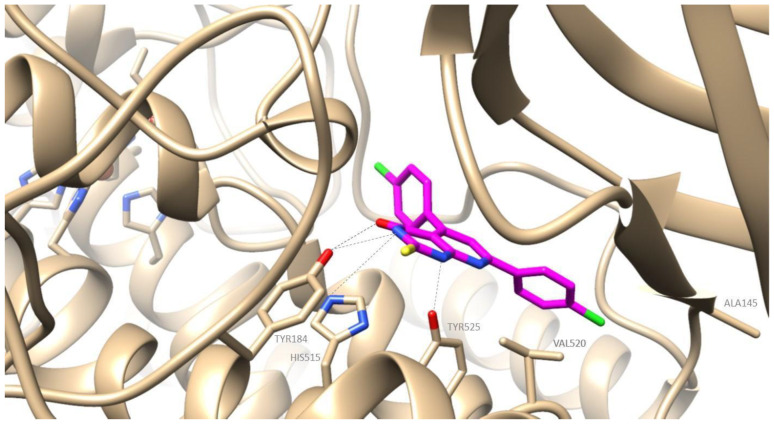
Preferred docking pose of **2f** (depicted in magenta) bound to soybean lipoxygenase (LOX-1).

The preferred docking poses of the most active derivatives, **2a**, **2f** and **1g**, are shown in Figure 4, Figure 5 and Figure 6 respectively. Pyrimidine derivatives **2a**, **2f** had an AutoDockVina score of −9.8 kcal/mol, while chalcone **1g** –8.9 kcal/mol binding to soybean LOX (PDB code: 3PZW). A one-to-one correlation is difficult to reach between the results obtained from the in vitro inhibition of soybean lipoxygenase that represents experimental values and docking scores based on algorithms and scoring function calculations. The putative preferred orientation of the ligand bound to the protein was assessed with docking simulations. It seems that the tested compounds interact with the SLOX through allosteric interactions. Chalcone **1g** presents hydrophobic interactions with Phe143, Phe144, Val520, Tyr525, Trp772 and hydrogen bonds with Phe144 (Figure 4). Derivative **2a** presents hydrophobic interactions with amino acids Val126, Tyr525, Lys526, Trp772; hydrogen bonds with Tyr525 and pi stacking interactions with Tyr525 (Figure 5). Derivative **2f** seems to develop hydrophobic interactions with Val520, Tyr525; hydrogen bonds with Tyr184, His515, Tyr525 and halogen bonds between chlorine atoms and Ala145 (Figure 6). Since most LOX inhibitors are antioxidants or free radical scavengers [40] and lipoxygenation occurs *via* a carbon-centered radical on a lipid chain, it is likely that compounds **2a**, **2f** and **1g** exert their activity by extending into the hydrophobic domain, blocking the approach of substrates to the active site and thus preventing oxidation by soybean LOX.

## 3. Experimental Section

### 3.1. Materials and Instruments

All chemicals, solvents, chemical and biochemical reagents were of analytical grade and purchased from commercial sources (Merck, Merck KGaA, Darmstadt, Germany, Fluka Sigma-Aldrich Laborchemikalien GmbH, Hannover, Germany, Alfa Aesar, Karlsruhe, Germany and Sigma St. Louis, MO, USA). Soybean lipoxygenase, sodium linoleate, arachidonic acid (AA) and 2,2-azinobis-2-methyl-propanimidamine HCl (AAPH) were obtained from Sigma Aldrich- Merck KGaA, Darmstadt, Germany. Nordihydroguaretic acid (NDGA), 1,1-diphenyl-2-picrylhydrazyl (DPPH), 6-Hydroxy-2,5,7,8-tetramethyl-chroman2-carboxylic acid (Trolox) and (2*S*)-2-amino-5-[[(2*R*)-1-(carboxymethylamino)-1-oxo-3-sulfanylpropan-2-yl]amino]-5-oxopentanoic acid (Glutathione) were purchased from the Sigma-Aldrich Chemical Co. (St. Louis, MO, USA). All starting materials were used without further purification. The used cell lines in our experiments were a kind offer from Associate Professor A.Pappa (Department of Molecular Biology & Genetics, Faculty of Health Sciences, Democritus University of Thrace, 68100 Alexandroupolis, Greece): (I) The human cancer cell lines A549 (non-small cell lung adenocarcinoma, ATCC CCL-185), were obtained from the American Type Culture Collection (Rockville, MD, USA) and (II) The human immortalized keratinocyte (HaCaT) cell line was kindly provided by Dr Sharon Broby (Dermal Toxicology & Effects Group; Centre for Radiation, Chemical and Environmental Hazards; Public Health England, Didcot, UK.

Melting points (uncorrected) were determined on a MEL-Temp II (Lab. Devices, Holliston, MA, USA). For the in vitro tests, UV-Vis spectra were obtained on a Perkin-Elmer 554 double-beam spectrophotometer (Perkin-Elmer Corporation Ltd., Lane Beaconsfield, Bucks, UK). Infrared spectra (KBr pellets) were recorded with a Perkin-Elmer 597 spectrophotometer (Perkin-Elmer Corporation Ltd., Lane Beaconsfield, Bucks, UK).

The ^1^H Nucleic Magnetic Resonance (^1^H-NMR) spectra were recorded at 500 MHz in CDCl_3_ or DMSO on an Agilent 500/54 (DD2) using tetramethylsilane as an internal standard unless otherwise stated. Agilent 500/54 (DD2) ^13^C-NMR spectra were obtained at 125 MHz in CDCl_3_ or DMSO solutions with tetramethylsilane as the internal reference unless otherwise stated. Chemical shifts are expressed in ppm and coupling constants *J* in Hz. Mass spectra were determined on a LC-PDA-MS Thermo Finnigan system (LC Pump Plus, Autosampler, Surveyor PDA Plus Detector) interfaced with an ESI MSQ PlusThermo Finnigan-Thermo Fisher Scientific Inc. Waltham, MA, USA, using MeOH as the solvent. Reactions were monitored by thin-layer chromatography on 5554 F_254_ Silica gel/TLC cards (Merck and Fluka Chemie GmbH Buchs, Steinheim, Switzerland). For preparative thin-layer chromatography (prep TLC) Silica gel 60 F_254_, plates 2 mm, Merck KGaA ICH078057 Darmstadt, Germany were used. For the experimental determination of the lipophilicity using reverse phase thin-layer chromatography (RP-TLC), TLC-Silica gel 60 F_254_ DC Kieselgel, Merck (20 × 20 cm), Merck KGaA, Darmstadt, Germany plates were used. For the purification of the products with liquid chromatography, silica gel 230–400 mesh Merck (Merck, Merck KGaA, Darmstadt, Germany) was used.

### 3.2. Chemistry General Procedure

#### 3.2.1. Synthesis of *α,β*-Unsaturated Ketones (**1a**–**1h**)

In a round-bottomed flask containing absolute ethanol (4 mL), placed in an ice bath, an appropriately substituted ketone (1.0 eq.) was added. Then, aqueous KOH 40% *w/v* (4 mL) was added dropwise. After 10 min of stirring, an appropriately substituted aldehyde (1.0 eq.) was added. The mixture was stirred in ice bath for 3 h. Upon completion of the reaction (TLC monitoring), the mixture was acidified with HCl 10% *w/v* [34]. The solid formed was subsequently collected by vacuum filtration, washed with water, dried and recrystallized from absolute ethanol.

All the synthesized *α,β*-unsaturated ketones have been reported in the literature [51,52,53,54,55,56,57].

*(E)*-1,3-diphenylprop-2-en-1-one (**1a**): Yield: 64%, yellowish solid, m.p.: 53–55 °C. ^1^H-NMR (500 MHz, DMSO-d_6_) δ 8.18–8.14 (m, 2H), 7.94 (d, *J* = 15.7 Hz, 1H), 7.89 (dd, *J* = 6.4, 2.9 Hz, 2H), 7.75 (d, *J* = 15.7 Hz, 1H), 7.68 (t, *J* = 7.3 Hz, 1H), 7.58 (t, *J* = 7.6 Hz, 2H), 7.49–7.44 (m, 3H). The spectral data were in agreement with the literature data [51].

*(E)*-1-(4-chlorophenyl)-3-phenylprop-2-en-1-one (**1b**): Yield: 69%, yellowish solid, m.p.: 96–98 °C. ^1^H-NMR (500 MHz, DMSO-d_6_) δ 8.19 (d, *J* = 8.4 Hz, 2H), 7.94 (d, *J* = 15.6 Hz, 1H), 7.90 (dd, *J* = 6.4, 3.3 Hz, 2H), 7.76 (d, *J* = 15.6 Hz, 1H), 7.65 (d, *J* = 8.4 Hz, 2H), 7.49–7.45 (m, 3H). The spectral data were in agreement with the literature data [52].

*(E)*-1-phenyl-3-(thiophen-2-yl)prop-2-en-1-one (**1c**): Yield: 96%, beige solid, m.p.: 58–60 °C. ^1^H-NMR (500 MHz, DMSO-d_6_): δ 8.11–8.06 (m, 2H), 7.92 (d, *J* = 15.4 Hz, 1H), 7.79 (d, *J* = 5.0 Hz, 1H), 7.70 (d, *J* = 3.8 Hz, 1H), 7.59–7.54 (m, 3H), 7.57 (d, *J* = 15.4 Hz, 1H), 7.23–7.17 (m, 1H). The spectral data were in agreement with the literature data [53].

*(E)*-1-(4-chlorophenyl)-3-(thiophen-2-yl)prop-2-en-1-one (**1d**): Yield: 54%, beige solid, m.p.: 122–124 °C. ^1^H-NMR (500 MHz, DMSO-d_6_): δ 8.11 (d, *J* = 8.6 Hz, 2H), 7.93 (d, *J* = 15.3 Hz, 1H), 7.81 (d, *J* = 5.0 Hz, 1H), 7.71 (d, *J* = 3.5 Hz, 1H), 7.62 (d, *J* = 8.6 Hz, 2H), 7.55 (d, *J* = 15.3 Hz, 1H), 7.20 (dd, *J* = 5.0, 3.5 Hz, 1H). The spectral data were in agreement with the literature data [54].

*(E)*-3-(4-chlorophenyl)-1-phenylprop-2-en-1-one (**1e**): Yield: 76%, yellowish solid, m.p.: 113–115 °C. ^1^H-NMR (500 MHz, CDCl_3_): δ 8.02 (d, *J* = 7.0 Hz, 2H), 7.76 (d, *J* = 15.7 Hz, 1H), 7.64–7.55 (m, 1H), 7.58 (d, *J* = 8.4 Hz, 2H), 7.54–7.49 (m, 2H), 7.51 (d, *J* = 15.7 Hz, 1H), 7.40 (d, *J* = 8.4 Hz, 2H). The spectral data were in agreement with the literature data [55].

*(E)*-1,3-bis(4-chlorophenyl)prop-2-en-1-one (**1f**): Yield: 67%, yellowish solid, m.p.: 160–162 °C. ^1^H-NMR (500 MHz, CDCl3): δ 7.96 (d, *J* = 8.6 Hz, 2H), 7.76 (d, *J* = 15.7 Hz, 1H), 7.58 (d, *J* = 8.6 Hz, 2H), 7.48 (d, *J* = 8.6 Hz, 2H), 7.46 (d, *J* = 15.7 Hz, 1H), 7.40 (d, *J* = 8.6 Hz, 2H). The spectral data were in agreement with the literature data [56].

*(E)*-3-(2,4-dichlorophenyl)-1-phenylprop-2-en-1-one (**1g**): Yield: 32%, white solid, m.p.: 68–70 °C. ^1^H-NMR (500 MHz, DMSO-d_6_): δ 8.28 (d, *J* = 8.6 Hz, 1H), 8.18 (dd, *J* = 8.4, 1.3 Hz, 2H), 8.05 (d, *J* = 15.6 Hz, 1H), 7.97 (d, *J* = 15.6 Hz, 1H), 7.77 (d, *J* = 2.2 Hz, 1H), 7.70 (t, *J* = 7.3 Hz, 1H), 7.61–7.58 (m, 2H), 7.57 (dd, *J* = 8.6, 2.2 Hz, 1H). The spectral data were in agreement with the literature data [55].

*(E)*-1-(4-chlorophenyl)-3-(2,4-dichlorophenyl)prop-2-en-1-one (**1h**): Yield: 29%, white solid, m.p.: 118–120 °C. ^1^H-NMR (500 MHz, DMSO-d_6_): δ 8.28 (d, *J* = 8.5 Hz, 1H), 8.20 (d, *J* = 8.7 Hz, 2H), 8.04 (d, *J* = 15.6 Hz, 1H), 7.97 (d, *J* = 15.6 Hz, 1H), 7.77 (d, *J* = 2.1 Hz, 1H), 7.66 (d, *J* = 8.7 Hz, 2H), 7.57 (dd, *J* = 8.5, 2.1 Hz, 1H). The spectral data were in agreement with the literature data [57].

#### 3.2.2. Synthesis of pyrido [2,3-*d*]pyrimidin-4(1*H*)-Ones (**2a**–**2h**)

In a round-bottomed flask, glacial acetic acid (3 mL), 4-amino-6-hydroxy-2-mercaptopyrimidine monohydrate (1.0 eq.) and appropriately substituted *α,β*-unsaturated ketone (1.0 eq.) were consecutively added. The mixture was refluxed for 96 h. Upon consumption of the starting material (TLC monitoring), the mixture was poured into crushed ice and left to melt. The precipitated solid was filtered and washed with a saturated solution of NaHCO_3_ (15 mL). Then, extraction with ethyl acetate (3 × 15 mL) followed and the mixture was washed with brine (1 × 15 mL). The organic layer was collected, dried over anhydrous Na_2_SO_4_ and concentrated under reduced pressure. The crude product was purified by column chromatography, preparative thin-layer chromatography or recrystallized from the appropriate solvent.

Compounds **2a**, **2b**, **2d**, **2e** and **2f** have been previously reported in the literature, whereas compounds **2c, 2g** and **2h** are novel derivatives (Figure S1, Supplementary Material).

5,7-diphenyl-2-thioxo-2,3-dihydropyrido [2,3-*d*]pyrimidin-4(1*H*)-one (**2a**): The crude product was purified by column chromatography (1% *v/v* MeOH/CHCl_3_). Yield: 51%, yellow solid, m.p.: > 300 °C. ^1^H-NMR (500 MHz, DMSO-d_6_) δ 13.08 (br s, 1H), 12.36 (br s, 1H), 8.26–8.21 (m, 2H), 7.64 (s, 1H), 7.55–7.51 (m, 3H), 7.47–7.40 (m, 5H). The spectral data were in agreement with the literature data [35].

7-(4-chlorophenyl)-5-phenyl-2-thioxo-2,3-dihydropyrido [2,3-*d*]pyrimidin-4(1*H*)-one (**2b**): The crude product was purified by column chromatography (1% *v/v* MeOH/CHCl_3_). Yield: 65%, yellowish solid, m.p.: > 300 °C. ^1^H-NMR (500 MHz, DMSO-d_6_): δ 13.10 (br. s, 1H), 12.37 (br. s, 1H), 8.28 (d, *J* = 8.7 Hz, 2H), 7.68 (s, 1H), 7.61 (d, *J* = 8.7 Hz, 2H), 7.47–7.41 (m, 5H). The spectral data were in agreement with the literature data [33].

7-phenyl-5-(thiophen-2-yl)-2-thioxo-2,3-dihydropyrido [2,3-*d*]pyrimidin-4(1*H*)-one (**2c**): The crude product was recrystallized from ethanol. Yield: 17%, brown solid, m.p.: 260–262 °C. IR (KBr, cm^−1^): 3061.5, 2907.6, 1691.4, 1555.1, 1170.9. ^1^H-NMR (500 MHz, DMSO-d_6_): δ 13.07 (br. s, 1H), 12.43 (br. s, 1H), 8.22 (dd, *J* = 6.4, 3.1 Hz, 2H), 7.77 (d, *J* = 5.0 Hz, 1H), 7.74 (s, 1H), 7.57–7.52 (m, 3H), 7.48 (d, *J* = 3.6 Hz, 1H), 7.17–7.14 (m, 1H). ^13^C-NMR (125 MHz, DMSO-d_6_): δ 175.3, 159.4, 158.7, 152.9, 146.2, 138.1, 136.3, 130.9, 130.2, 128.9, 128.8, 127.6, 126.9, 119.4, 107.6. LC-MS (ESI, *m/z*, MeOH): [M – H]^−^ = 336, [M + Na]^+^ = 360, [M + K]^+^ = 376, [M + MeOH + Na]^+^ = 392. Elemental Analysis C, H, N. Calculated % (C_17_H_11_N_3_OS_2_): C: 60.51, H: 3.29, N: 12.46. Found % (C_17_H_11_N_3_OS_2_): 65.95, H: 3.31, N: 12.48.

7-(4-chlorophenyl)-5-(thiophen-2-yl)-2-thioxo-2,3-dihydropyrido [2,3-*d*]pyrimidin-4(1*H*)-one (**2d**): The crude product was recrystallized from ethyl acetate. Yield: 14%, yellowish solid, m.p.: > 300 °C. ^1^H-NMR (500 MHz, DMSO-d_6_): δ 13.08 (br. s, 1H), 12.44 (br. s, 1H), 8.26 (d, *J* = 8.7 Hz, 2H), 7.79–7.77 (m, 1H), 7.76 (s, 1H), 7.61 (d, *J* = 8.7 Hz, 2H), 7.48 (dd, *J* = 3.6, 1.3 Hz, 1H), 7.16 (dd, *J* = 5.1, 3.6 Hz, 1H). The spectral data were in agreement with the literature data [36].

5-(4-chlorophenyl)-7-phenyl-2-thioxo-2,3-dihydropyrido [2,3-*d*]pyrimidin-4(1*H*)-one (**2e**): The crude product was recrystallized from ethanol. Yield: 18%, yellowish solid, m.p.: > 300 °C. ^1^H-NMR (500 MHz, DMSO-d_6_): δ 13.10 (br. s, 1H), 12.39 (br. s, 1H), 8.27–8.22 (m, 2H), 7.67 (s, 1H), 7.57–7.52 (m, 3H), 7.51–7.46 (m, 4H). The spectral data were in agreement with the literature data [37].

5,7-bis(4-chlorophenyl)-2-thioxo-2,3-dihydropyrido [2,3-*d*]pyrimidin-4(1*H*)-one (**2f**): The crude product was purified by preparative thin-layer chromatography (1% *v/v* MeOH/CHCl_3_). Yield: 20%, off-white solid, m.p.: > 300 °C. ^1^H-NMR (500 MHz, CDCl_3_**):** δ 9.53 (br. s, 1H), 9.04 (br. s, 1H), 8.02 (d, *J* = 8.6 Hz, 2H), 7.50 (d, *J* = 8.7 Hz, 2H), 7.47–7.43 (m, 2H), 7.46 (s, 1 H), 7.33 (d, *J* = 8.6 Hz, 2H). The spectral data were in agreement with the literature data [33].

5-(2,4-dichlorophenyl)-7-phenyl-2-thioxo-2,3-dihydropyrido [2,3-*d*]pyrimidin-4(1*H*)-one (**2g**): The crude product was purified by column chromatography (1% *v/v* MeOH/CH_2_Cl_2_). Yield: 16%, off-white solid, m.p.: > 300 °C. IR (KBr, cm^−1^): 3064.4, 2919.1, 1679.9, 1555.9, 1176.8. ^1^H-NMR (500 MHz, DMSO-d_6_): δ 13.22 (br. s, 1H), 12.48 (br. s, 1H), 8.25 (dd, *J* = 6.7, 2.9 Hz, 2H), 7.74 (s, 1H), 7.71 (d, *J* = 2.0 Hz, 1H), 7.57–7.52 (m, 3H), 7.53 (dd, *J* = 8.2, 2.0 Hz, 1H), 7.42 (d, *J* = 8.2 Hz, 1H). ^13^C-NMR (125 MHz, DMSO-d_6_): δ 175.6, 160.1, 158.5, 149.2, 144.9, 136.7, 136.2, 133.4, 132.5, 131.1, 130.9, 128.9, 128.1, 127.6, 126.9, 118.5, 108.6. LC-MS (ESI, *m/z*, MeOH): [M – H]^−^ = 399, 401, 403 (9:6:1), [M + K]^+^ = 439, 441, 443 (9:6:1). Elemental Analysis C, H, N. Calculated % (C_19_H_11_Cl_2_N_3_OS): C: 57.01, H: 2.78, N: 10.5. Found % (C_19_H_11_Cl_2_N_3_OS): C: 57.41, H: 2.88, N: 10.60.

7-(4-chlorophenyl)-5-(2,4-dichlorophenyl)-2-thioxo-2,3-dihydropyrido [2,3-*d*]pyrimidin-4(1*H*)-one (**2h**): The crude product was purified by preparative thin-layer chromatography (1% *v/v* MeOH/CH_2_Cl_2_). Yield: 15%, off-white solid, m.p.: 274–276 °C. IR (KBr, cm^−1^): 3100.3, 2919.1, 1674.6, 1558.5, 1173.9. ^1^H-NMR (500 MHz, CDCl_3_): δ 9.78 (br. s, 1H), 9.27 (br. s, 1H), 8.02 (d, *J* = 8.7 Hz, 2H), 7.51 (d, *J* = 2.0 Hz, 1H), 7.49 (d, *J* = 8.7 Hz, 2H), 7.43 (s, 1H), 7.36 (dd, *J* = 8.2, 2.0 Hz, 1H), 7.18 (d, *J* = 8.2 Hz, 1H). ^13^C-NMR (125 MHz, CDCl_3_): δ 174.5, 160.9, 157.7, 151.8, 150.9, 138.1, 135.6, 135.3, 134.7, 133.2, 129.9, 129.6, 129.5, 129.1, 127.4, 119.4, 108.4. LC-MS (ESI, *m/z*, MeOH): [M – H]^−^ = 432, 434, 436, 438 (27:27:9:1). Elemental Analysis C, H, N. Calculated % (C_19_H_10_Cl_3_N_3_OS): C: 52.5, H: 2.32, N: 9.67. Found % (C_19_H_10_Cl_3_N_3_OS): C: 52.45, H: 2.29, N: 9.69.

### 3.3. Physicochemical Studies

#### Experimental Determination of Lipophilicity as R_M_ Values

Lipophilicity as R_M_ values was determined through reversed-phase TLC (RP-TLC) on silica gel plates impregnated with 5% *v/v* liquid paraffin in light petroleum ether. Methanol/water mixture (70/30, *v/v*) was used as mobile phase. Closed chromatography tanks saturated with the mobile phase at 24 °C were used for the development of the plates while the spots were detected under UV light. For the determination of R_M_ values, five individual measurements of R_f_ values were recorded, and the R_M_ values were derived from the following Equation (1) [40,49].

(1) RM=log1Rf−1

### 3.4. Biological In Vitro Assays

A stock DMSO solution (10 mM) of the synthesized compounds was prepared for the in vitro assays 3.4.1, 3.4.2, 3.4.3 and 3.4.4, from which several dilutions were made with the appropriate buffer. Each experiment was performed in triplicate and the standard deviation of the absorbance was less than 10% of the mean.

#### 3.4.1. Determination of the Reducing Activity of the Stable Radical DPPH

In vitro study was performed as reported previously by our group. In this experiment, 20 μL of the tested compound (from the stock solution), 980 μL EtOH and 1 mL ethanolic solution of DPPH (100 μM) were added in a testing tube in a final volume of 2 mL. The free radical scavenging ability was evaluated by recording the change of absorbance at 517 nm, produced by the reduction of DPPH, after 20 and 60 min at room temperature. NDGA was used as the reference. The results are presented in Table 4 [40,49].

#### 3.4.2. Inhibition of AAPH Induced Linoleic Acid Peroxidation

In vitro study was performed as reported previously by our group. AAPH was used as a free radical generator of alkyl peroxyl radicals. For this experimental procedure, 10 μL of the tested compound (from the stock solution), 10 μL sodium linoleate (16 mM), 50 μL AAPH (40 mM) and 930 μL phosphate-buffered solution (pH 7.4) were added in a UV cuvette (1 mL), pre thermostated at 37 °C. The ability of the compounds to prevent the oxidation of linoleic acid from alkyl peroxyl radicals was evaluated by recording the change of absorbance at 234 nm, due to the conversion of linoleic acid to 13-hydroperoxy-linoleic acid. Trolox was used as the reference. The results are presented in Table 4 [40,47].

#### 3.4.3. Inhibition of Soybean Lipoxygenase

In vitro study was performed as reported previously by our group. In this experiment, 10 μL of the tested compound (from the stock solution) were incubated with 100 μL sodium linoleate (diluted in Tris buffer), 200 μL LOX solution (1:9 × 10^−4^ *w/v* in saline) and 690 μL Tris buffer (pH 9.0) at room temperature. The absorbance was measured at 234 nm, where the change of absorbance was recorded due to the formation of 13-hydroperoxy-linoleic acid. NDGA was used as the reference. In order to determine the IC_50_ values, different concentrations were used. The results are presented in Table 4 [40,47,49].

#### 3.4.4. Interaction with Glutathione

In vitro study was performed as reported previously by our group. The stock solution of the tested compound was diluted with phosphate buffer (pH 7.4). The concentration of the solution was chosen in order that the absorbance maximum was included between 0.5 and 1. The solution was incubated at 37 °C for 24 h. The absorbance of the solution was measured at λ_max_ of each compound and the respective ε_λmax_ value was determined. The error limits of ε values did not exceed 2%.

The experiment was repeated with the addition of glutathione in two-fold and ten-fold concentrations, respectively, compared to the concentration of the tested compound. The molar absorption coefficient, ε, was calculated using the Beer–Lambert law, as shown in Equation (2):

(2) A=ε×c×d

where A is the absorbance, ε is the molar absorption coefficient (M^−1^ cm^−1^), c is the concentration (M) and d is the pathlength (cm). The results are presented in Table 5 [58].

#### 3.4.5. Cell Viability and Cytotoxicity Assay

Τhe MTT assay was used to evaluate cell viability or cell toxicity, depending on the cell line being tested. The experimental procedure was performed in a 96-well plate, where the cells were seeded and incubated (37 °C, 5% CO_2_) for 24 h to grow and cover the surface of the wells. Τhe seeding concentrations for the HaCaT and A549 cells were 30 × 10^4^ cells/mL and 20 × 10^4^ cells/mL, respectively [59]. After the first incubation, the solutions of the compounds (with different concentrations) were added to the adherent cells and incubated for another 24 h. The compounds were tested in triplicate against the control (only cells in medium), the blank (only medium) and the positive/negative control (cells in 100 μM silibinin solution) triplicates. Silibinin, a flavonoid with known anticancer activities, was used as a negative control for the cell viability assay and as positive control for the cytotoxicity testing assay [60]. The light-sensitive MTT-standard solution was prepared by dissolving 1 mg/mL MTT in PBS and then filtrated (in a 0.22 mm filter (Millipore, Carrigtwohill, Ireland)). On the third day, an appropriate amount of ΜΤΤ solution was added to each well and the plate was incubated for 4 h. During the incubation time, the formazan crystals were formed, which were then dissolved with 100 μL DMSO per well. Finally, the plate was stirred for 30 min, and the optical density (OD) was measured in a plate reader spectrophotometer at 450 nm.

The average of the absorbances of each triplicate was calculated and the results of cell viability and cytotoxicity rates (%) were obtained via the following Equations (3) and (4):

(3) $$\%Viability=\left(\frac{\left(OD\;Control\;at\;540\;nm-Blank\right)-\left(OD\;sample\;at\;540\;nm-Blank\right)}{\left(OD\;Control\;at\;540\;nm-Blank\right)}\right)\times 100$$

(4) $$\%Toxicity=\left(\frac{\left(OD\;sample\;at\;540\;nm-Blank\right)-\left(OD\;Control\;at\;540\;nm-Blank\right)}{\left(OD\;Control\;at\;540\;nm-Blank\right)}\right)\times 100$$

### 3.5. Computational Methods

#### 3.5.1. Molecular Docking Studies on Soybean Lipoxygenase

UCSF Chimera [61] (Resource for Biocomputing, Visualization, and Informatics at the University of California, San Francisco) was used for the visualization of the protein (PDB code: 3PZW). Water molecules were removed, missing residues were added with Modeller (10.3) [62] (Departments of Biopharmaceutical Sciences and Pharmaceutical Chemistry, and California Institute for Quantitative Biomedical Research, Mission Bay Byers Hall, University of California San Francisco, San Francisco, CA 94143, USA), hydrogen atoms and AMBER99SB-ILDN charges were added and the charge on iron was set to +2.0 with no restraint applied to the iron atom and the ligands. Open Babel (3.1.1) [63] was used to generate and minimize ligand 3D coordinates using the MMFF94 [64] force field. Ligand topologies and parameters were generated by ACPYPE (2021.12.24) [65] using AnteChamber (AmberTools 22.10) [66]. Energy minimizations were carried out using the AMBER99SB-ILDN [67] force field with GROMACS (4.6.5) [68]. Docking was performed with AutoDock Vina (Vina 1.2.3; https://vina.scripps.edu/, (accessed on 1 March 2023)) [69] by applying a grid box of size 100 Å, 70 Å and 70 Å in the X, Y, and Z dimensions, respectively. The generation of docking input files and the analysis of the docking results were accomplished with UCSF Chimera. Docking was carried out with an exhaustiveness value of 10 and a maximum output of 20 docking modes.

#### 3.5.2. In Silico Determination of Drug-Likeness

Lipinski’s rule of five is usually performed to evaluate drug-likeness [70]. Thus, compounds were subjected to molecular properties prediction in the online software Molinspiration (https://www.molinspiration.com/, (accessed on 1 March 2023)).

## 4. Conclusions

In conclusion, eight pyrido [2,3-*d*]pyrimidine derivatives, from which **2c**, **2g** and **2h** are presented for the first time in the literature, were synthesized, spectroscopically confirmed and evaluated for their biological activities. The corresponding chalcones were also synthesized as starting materials. In the DPPH assay, the synthesized compounds showed low to moderate reducing activity. However, the majority of the compounds presented high lipid peroxidation inhibitory activity, with the most potent being pyrimidine derivatives **2g** and **2h**. Lipophilicity seems to favor anti-lipid peroxidation activity. Moreover, derivatives **2a** and **2f** were found to be the most potent LOX inhibitors with IC_50_ values of 42 μM and 47.5 μM, respectively. Chalcone **1g** was also assessed with high anti-LOX activity (IC_50_ = 17 μM). The molecular docking studies revealed that compounds **2a**, **2f** and **1g** interact in an allosteric mode with the enzyme and they probably exert their activity by extending into the hydrophobic domain and blocking the substrates to the binding site, hence preventing oxidation. All the compounds, except for **1h**, seem to conjugate with glutathione. In the MTT assay, none of the synthesized analogues showed cell viability at 100 μM regarding the HaCaT cell line. Some of them presented moderate viability at 50 μM, which further increased at 10 μΜ. Derivative **2f** showed viability only at 1 μM, with a percentage of 73.4%. In the case of adenocarcinoma epithelial cells (A549), most compounds showed strong cell toxicity at 100 μM, whereas their effect was moderate to high at 75 μΜ and 50 μM, with derivative **2d** showing the strongest cytotoxic effects (100%). Finally, moderate cytotoxicity effects were obtained from treating cells with a concentration of 10 μM. It is important to mention that the pyrimidine derivatives **2a** and **2f** were the most potent LOX inhibitors, could conjugate with glutathione and presented cytotoxicity towards adenocarcinoma epithelial cells (A549). Thus, they could be further investigated as multifunctional molecules.

## Figures and Tables

**Table 1:** Substituents of pyrimidine derivatives.

Compound	Pyrimidine Scaffold	Ar_1_	Ar_2_
**2a**	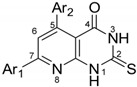	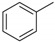	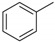
**2b**		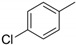	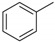
**2c**		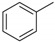	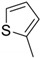
**2d**		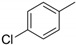	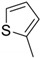
**2e**		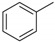	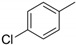
**2f**		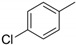	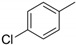
**2g**		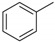	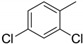
**2h**		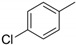	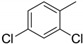

**Table 2:** Lipophilicity values of the synthesized compounds. Experimental R_M_ values. Theoretically calculated clog *P* values calculated using the Bio-Loom Program, Biobyte.

Compound	R_M_	clog *P*	Compound	R_M_	clog *P*
**1a**	−0.57	3.08	**2a**	−0.95	3.64
**1b**	−0.50	3.87	**2b**	−0.87	4.36
**1c**	−0.52	2.73	**2c**	−0.83	3.53
**1d**	−0.46	3.52	**2d**	−0.80	4.24
**1e**	−0.50	3.80	**2e**	−0.87	4.36
**1f**	−0.24	4.59	**2f**	−0.91	5.07
**1g**	−0.21	4.51	**2g**	−0.60	4.82
**1h**	−0.05	5.30	**2h**	−0.48	5.54

SD standard deviation < 10%.

**Table 3:** Drug-likeness studies of the synthesized compounds.

Compound	milog P ^a^	TPSA ^b^	No of Atoms	No of O and N ^c^	No of OH and NH ^d^	No of Violations	No of Rotational Bonds ^e^	Volume ^f^	MW ^g^
**1a**	3.81	17.07	16	1	0	0	3	201.85	208.26
**1b**	4.49	17.07	17	1	0	0	3	215.39	242.71
**1c**	3.53	17.07	15	1	0	0	3	192.56	214.29
**1d**	4.21	17.07	16	1	0	0	3	206.10	248.73
**1e**	4.49	17.07	17	1	0	0	3	215.39	242.71
**1f**	5.17	17.07	18	1	0	1	3	228.92	277.15
**1g**	4.92	17.07	18	1	0	0	3	228.92	277.15
**1h**	5.59	17.07	19	1	0	1	3	242.46	311.60
**2a**	3.69	61.55	24	4	2	0	2	283.51	331.40
**2b**	4.36	61.55	25	4	2	0	2	297.05	365.85
**2c**	3.47	61.55	23	4	2	0	2	274.22	337.43
**2d**	4.15	61.55	24	4	2	0	2	287.76	371.87
**2e**	4.36	61.55	25	4	2	0	2	297.05	365.85
**2f**	5.04	61.55	26	4	2	1	2	310.58	400.29
**2g**	4.97	61.55	26	4	2	0	2	310.58	400.29
**2h**	5.65	61.55	27	4	2	1	2	324.12	434.74

^a^ Logarithm of partition coefficient between n-octanol and water (milog P); ^b^ topological polar surface area (TPSA); ^c^ number of hydrogen bond acceptors; ^d^ number of hydrogen bond donors; ^e^ number of rotatable bonds; ^f^ molecular volume; ^g^ molecular weight.

**Table 4:** Interaction with the stable free radical DPPH, AAPH-induced linoleic acid peroxidation inhibitory activity and soybean LOX in vitro inhibitory activity of the synthesized compounds *.

Compound	RA % 100 μM 20 min	RA % 100 μM 60 min	AAPH % Inhibition 100 μM	LOX % Inhibition 100 μM or IC_50_ (μM)
**1a**	22	no	6	26
**1b**	no	4	51	39
**1c**	17	no	no	6
**1d**	no	no	8	72.5 μM
**1e**	no	no	4	55.5 μM
**1f**	no	no	51	4
**1g**	2	8	39	17 μM
**1h**	no	no	2	no
**2a**	29	56	52	42 μM
**2b**	no	1	62	6
**2c**	14	10	52	68.5 μM
**2d**	no	no	62	70 μM
**2e**	24	42	33	76 μM
**2f**	no	no	no	47.5 μM
**2g**	1	no	95	54 μM
**2h**	no	no	99	27
**NDGA** **Trolox**	87 -	93 -	- 92	93 (0.45 μM) -

* no: no activity was determined under the experimental conditions.

**Table 5:** Interaction of the most active pyrimidine derivatives with glutathione.

Compound	λ_max_ ^#^	ε_λmax_ ^$^
**2a**	353 nm	5385
2a + 2[GSH] *	353 nm	4200
**2f**	358 nm	1130
**2f + 2[GSH]**	358 nm	220

* [GSH]: Glutathione concentration; ^#^ λ_max_: Wavelength corresponding to the maximum absorbance of the tested compound; ^$^ ε_λmax_: Molar absorption coefficient calculated for the λ_max_ of the tested compounds.

**Table 6:** HaCaT cell line viability (%) after the treatment with the compounds at the concentrations of 100 μΜ, 50 μM, 10 μM and 1 μM.

Compound	% Cell Viability			
	100 μΜ	50 μΜ	10 μΜ	1 μΜ
**1a**	no	-	55.1	-
**1b**	no	-	28.1	-
**1c**	no	61.7	100	-
**1d**	no	53	72.8	-
**1e**	no	89.9	100	-
**1f**	no	57	100	-
**1g**	2.1	32.7	100	-
**1h**	no	-	25.4	-
**2a**	no	85.6	100	-
**2b**	no	26	75.1	-
**2c**	no	-	16.1	-
**2d**	no	-	50	-
**2e**	no	-	22.9	-
**2f**	no	-	no	73.4
**2g**	no	-	56.7	-
**2h**	no	89.9	100	-
**Silibinin (negative control)**	14.9	-	-	-

no: no activity (viability) was determined under the experimental conditions.

**Table 7:** Evaluation of the tested compounds’ cytotoxicity (%) against A549 cell line, at the concentrations of 100 μΜ, 75 μM 50 μM and 10 μM.

Compound	% Cell Toxicity		
	100 μΜ	50 μΜ	10 μΜ
**1a**	100	81.6	41.8
**1b**	100	88.1	49.2
**1c**	8.8	no	
**1d**	100	83.2	47.4
**1e**	36.8	6.8	
**1f**	100	67.6	48.9
**1g**	10.8	no	no
**1h**	100	81.1	49.1
**2a**	80.9	75	56.4
**2b**	100	83.5	47.9
**2c**	100	63.6	49.4
**2d**	100	100	48.6
**2e**	82.1	83.9	48
**2f**	100	88.1	48.6
**2g**	79.4	79.1	
**2h**	100	76.9	49.1
**Silibinin (positive control)**	96.4		

no: no activity (cytotoxicity) was determined in our experimental conditions.

**Table 8:** Docking scores for blind docking expressed in kcal/mol for all the synthesized derivatives.

Compound	Docking Score (kcal/mol)
**1a**	−8.1
**1b**	−8.3
**1c**	−7.5
**1d**	−7.3
**1e**	−8.5
**1f**	−8.0
**1g**	−8.9
**1h**	−8.3
**2a**	−9.8
**2b**	−9.4
**2c**	−9.3
**2d**	−9.3
**2e**	−9.4
**2f**	−9.8
**2g**	−9.7
**2h**	−9.1

## Data Availability

Not applicable.

## Supplementary Materials

The following supporting information can be downloaded at: https://www.mdpi.com/article/10.3390/molecules28093913/s1, Figure S1: ^1^H-NMR and ^13^C-NMR data for the novel synthesized derivatives.

## Sample Availability

Samples of the all the synthesized compounds are available from the authors.
