# Supplementary material for: Novel Pyrimidine Derivatives as Antioxidant and Anticancer Agents: Design, Synthesis and Molecular Modeling Studies
Source: Molecules. 2023 May 5;28(9):3913. doi: 10.3390/molecules28093913 (PMC10180197; doi:10.3390/molecules28093913)
Supplement: Supplementary file 1 [file molecules-28-03913-s001.zip › molecules-2340957-SI.pdf]

Supplementary Materials

Novel Pyrimidine Derivatives as Antioxidant and Anticancer Agents: Design, Synthesis and Molecular Modeling Studies

Malama Myriagkou [1], Evangelia Papakonstantinou [2], Georgia-Eirini Deligiannidou [2], Alexandros Patsilinakos [3,†], Christos Kontogiorgis [2] and Eleni Pontiki [1,*]

[1] Department of Pharmaceutical Chemistry, School of Pharmacy, Faculty of Health Sciences, Aristotle University of Thessaloniki, 54124 Thessaloniki, Greece; myriagkou@pharm.auth.gr
[2] Laboratory of Hygiene and Environmental Protection, School of Medicine, Democritus University of Thrace, 25510 Alexandroupoli, Greece; evangeliapapakonstantinou7@gmail.com (E.P.); edeligia@med.duth.gr (G.-E.D.); ckontogi@med.duth.gr (C.K.)
[3] Department of Drug Chemistry and Technology, Sapienza University, 00185 Rome, Italy; alexandros.patsilinakos@gmail.com
* Correspondence: epontiki@pharm.auth.gr
† Current address: Sibylla Biotech S.p.A., 37121 Verona, Italy

Figure S1: ^{1}H-NMR and ^{13}C-NMR data for the novel synthesized derivatives

2c – ^{1}H-NMR

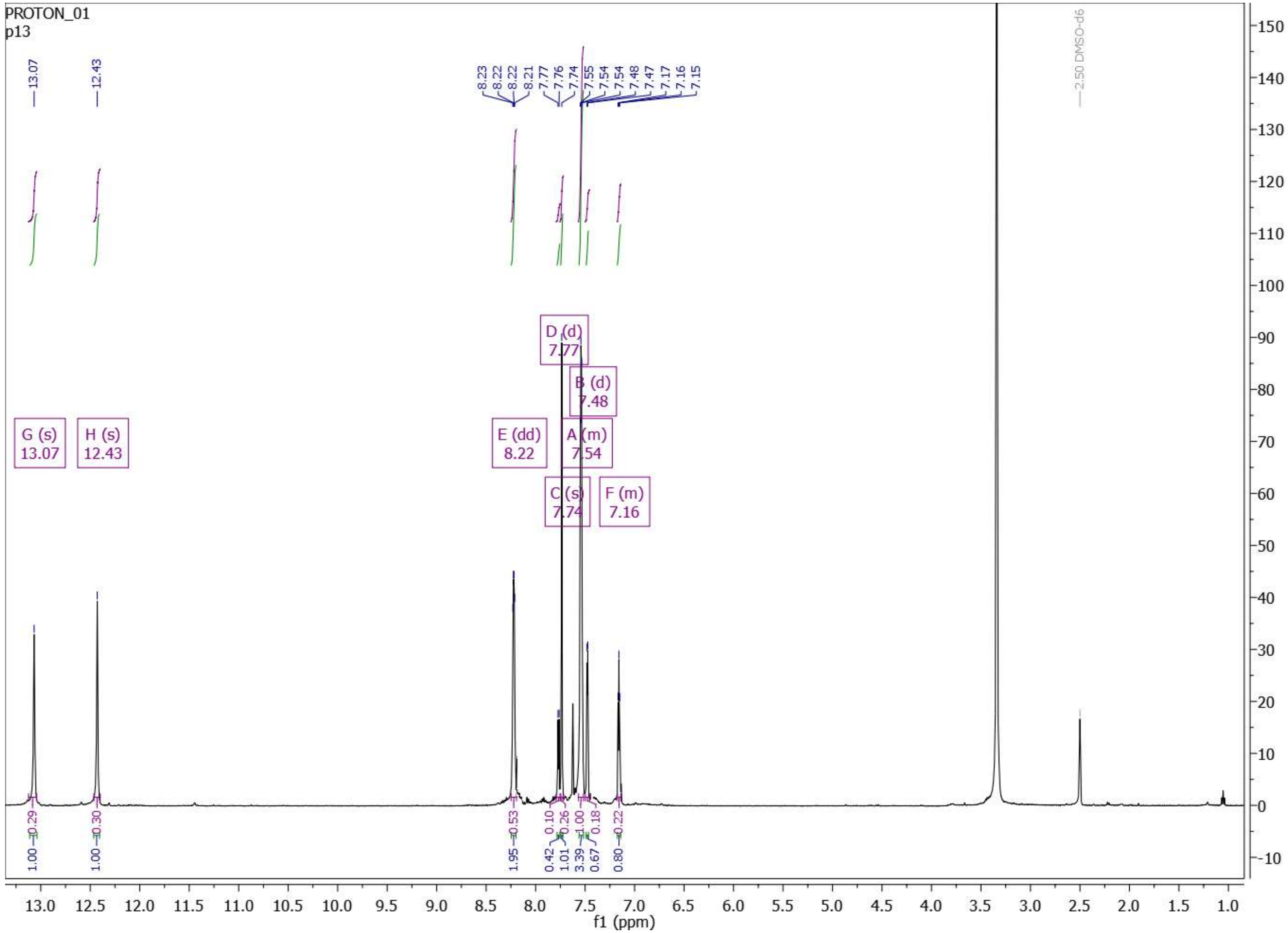

2c – ^{13}C-NMR

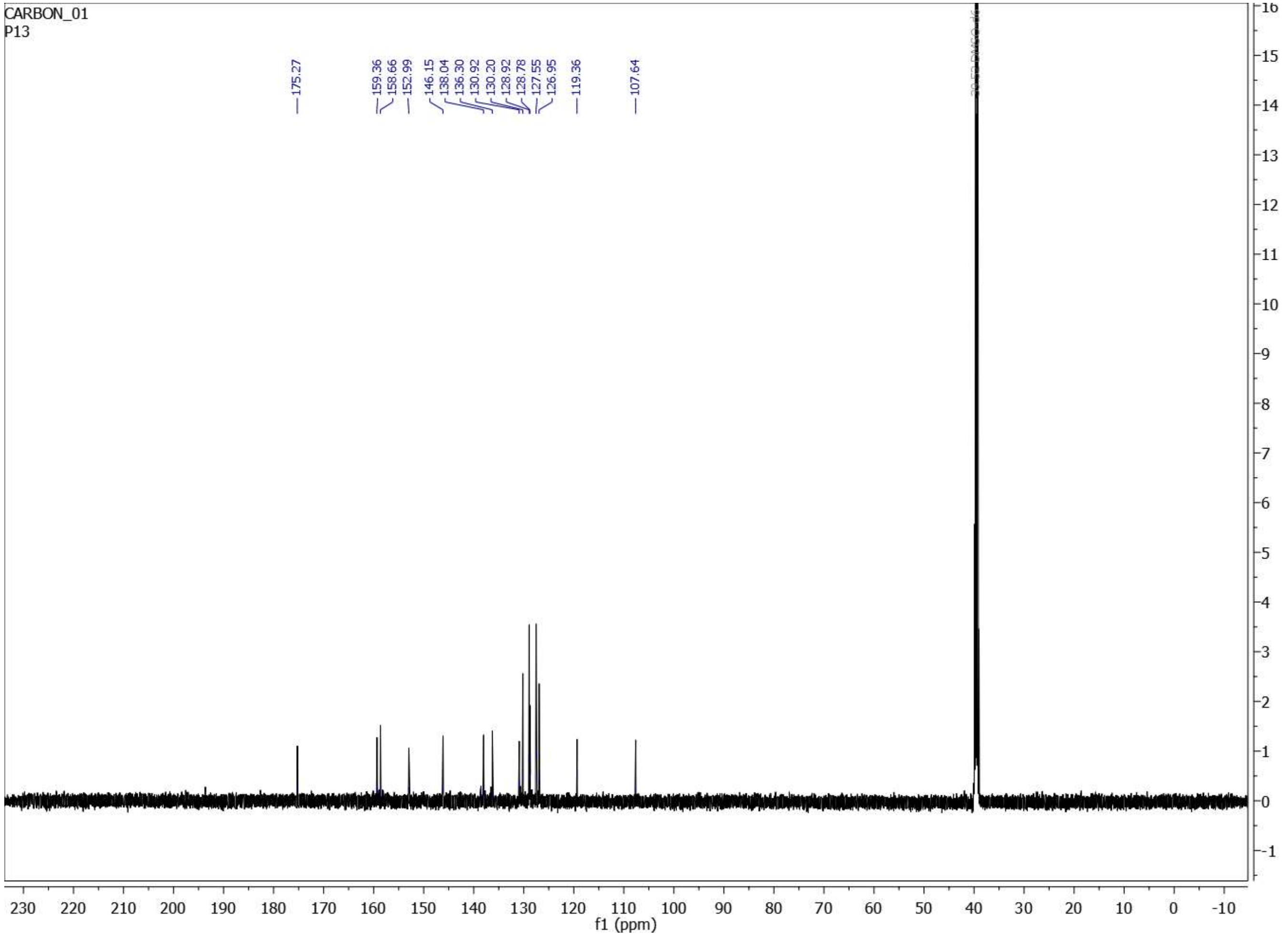
CARBON_01
P13
175.27
159.36
158.66
152.99
146.15
138.04
136.30
130.92
130.20
128.92
128.78
127.55
126.95
119.36
107.64
f1 (ppm)

2g - ^{1}H-NMR

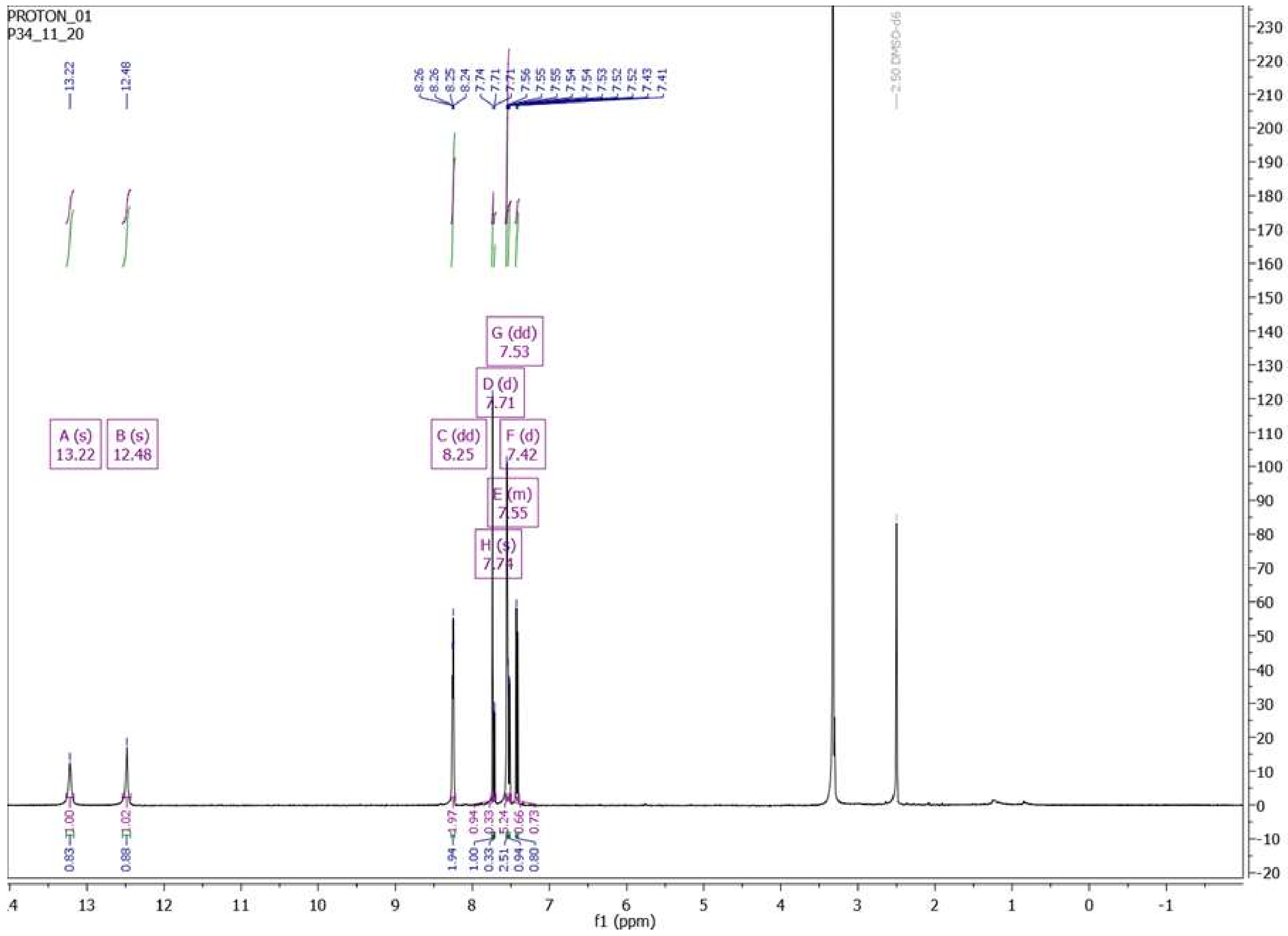
PROTON_01
P34_11_20
13.22
12.48
8.26
8.26
8.25
8.24
7.74
7.71
7.71
7.56
7.55
7.55
7.54
7.54
7.53
7.52
7.52
7.43
7.41
2.50 DMSO-d6
A (s)
13.22
B (s)
12.48
C (dd)
8.25
D (d)
7.71
G (dd)
7.53
F (d)
7.42
E (m)
7.55
H (s)
7.74
f1 (ppm)

2g – ^{13}C-NMR

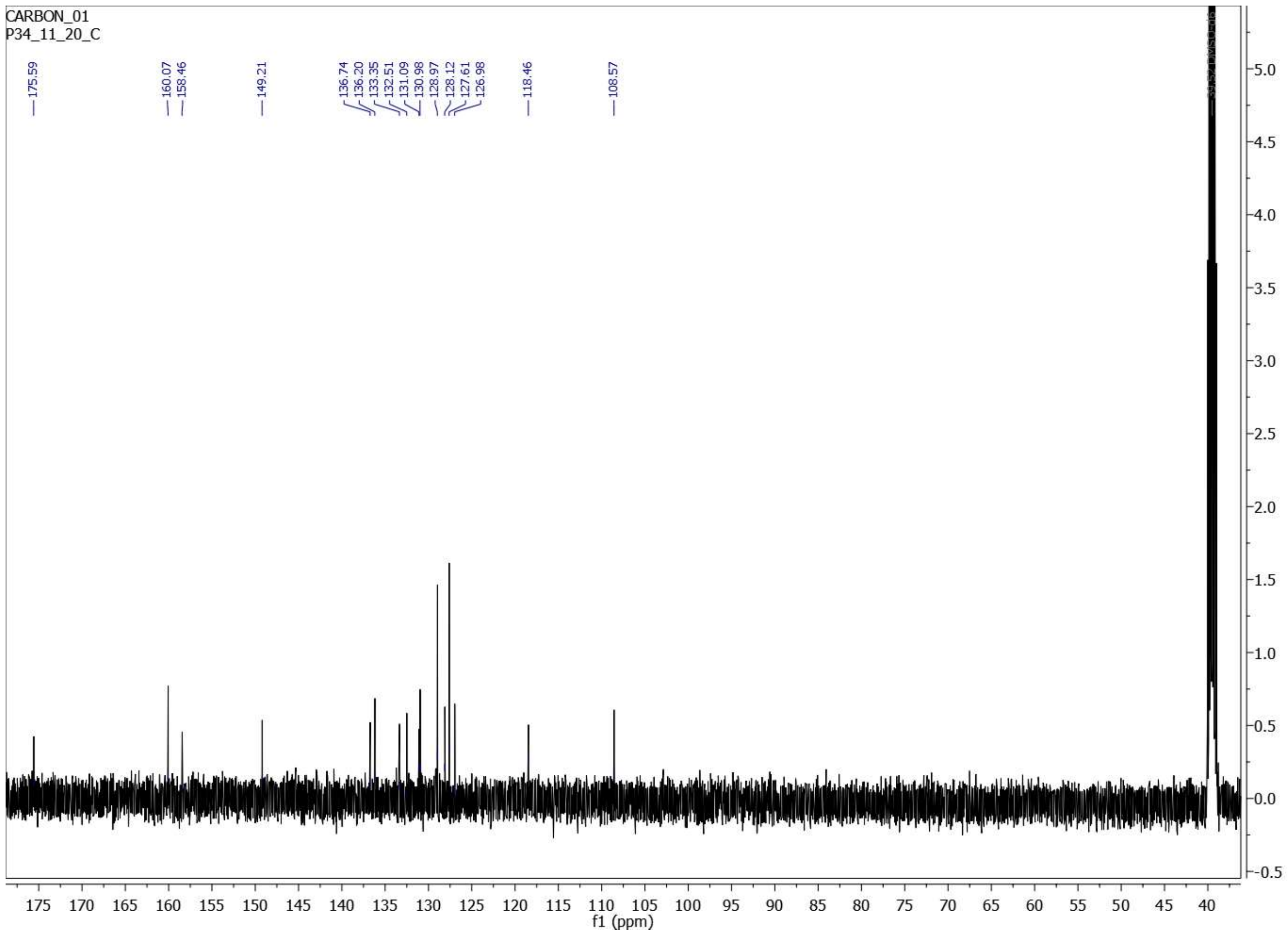

CARBON_01
P34_11_20_C
175.59
160.07
158.46
149.21
136.74
136.20
133.35
132.51
131.09
130.98
128.97
128.12
127.61
126.98
118.46
108.57
39.52 DMSO-d6
5.0
4.5
4.0
3.5
3.0
2.5
2.0
1.5
1.0
0.5
0.0
-0.5
175 170 165 160 155 150 145 140 135 130 125 120 115 110 105 100 95 90 85 80 75 70 65 60 55 50 45 40
f1 (ppm)

2h – ^{1}H-NMR

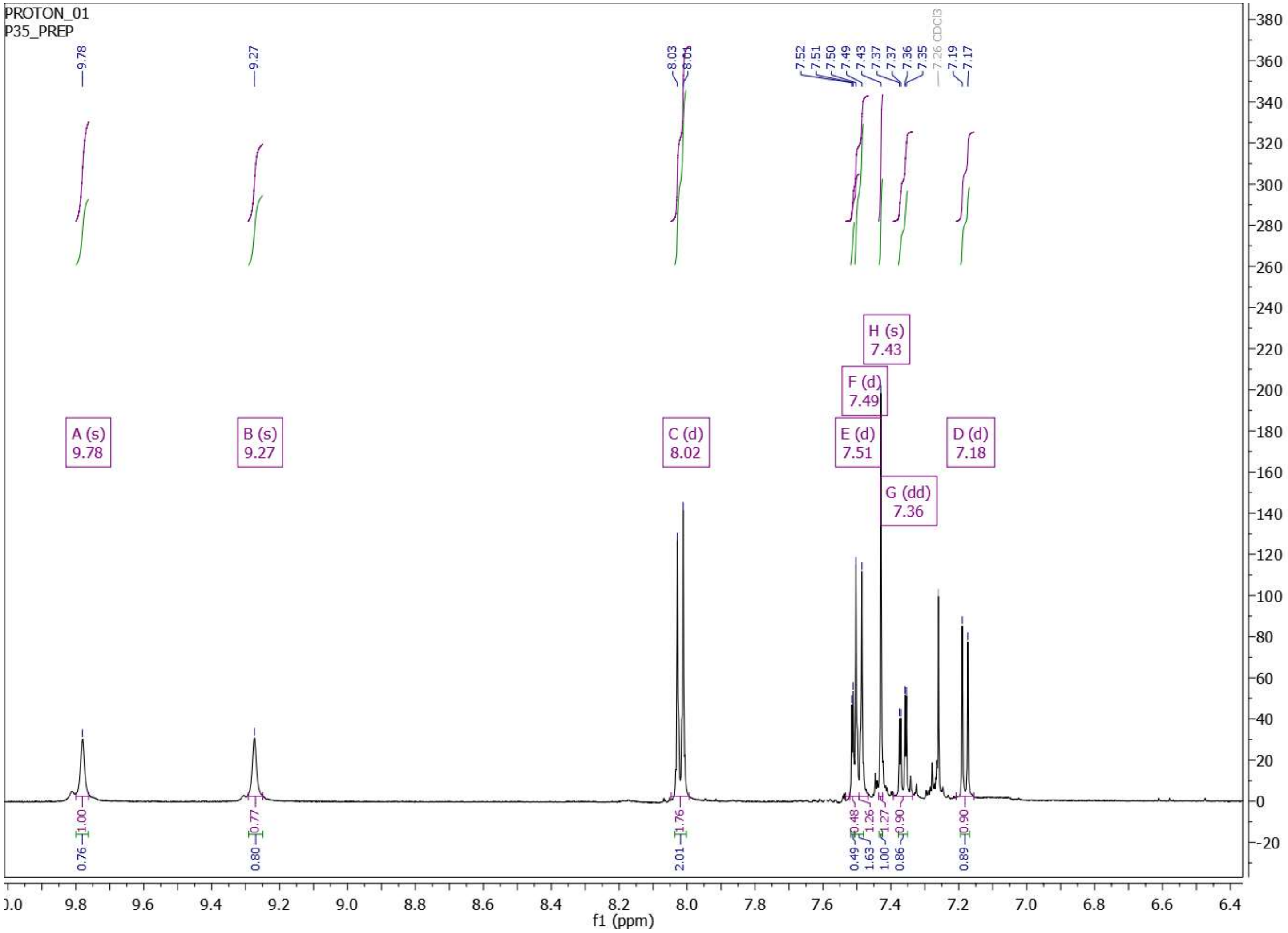
PROTON_01
P35_PREP
A (s)
9.78
B (s)
9.27
C (d)
8.02
E (d)
7.51
F (d)
7.49
H (s)
7.43
G (dd)
7.36
D (d)
7.18
7.26 CDCl3
f1 (ppm)

2h – ^{13}C-NMR

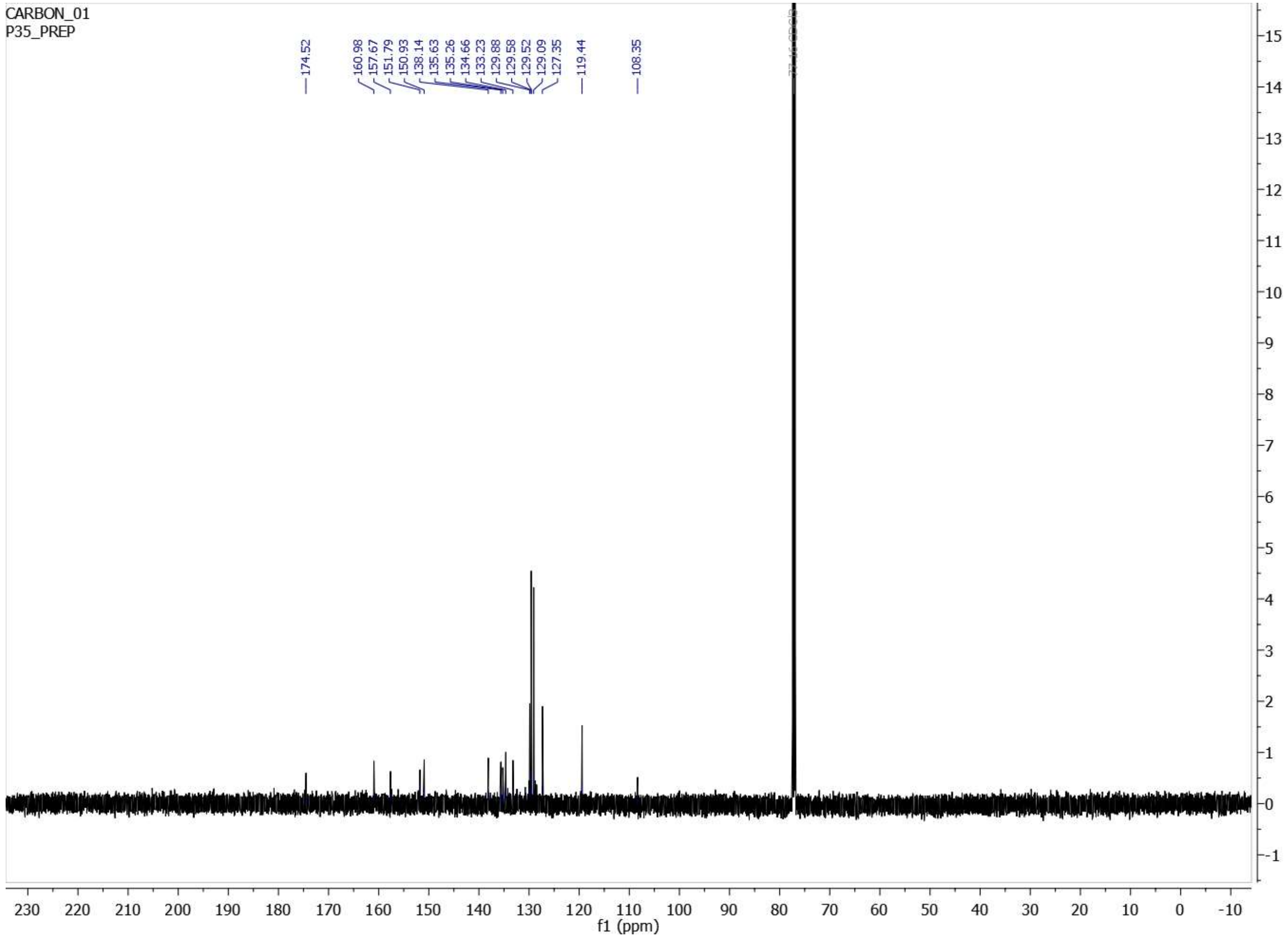
CARBON_01
P35_PREP
174.52
160.98
157.67
151.79
150.93
138.14
135.63
135.26
134.66
133.23
129.88
129.58
129.52
129.09
127.35
119.44
108.35
f1 (ppm)
